# Traumatic brain injury alters presentation of mild behavioral impairment domains across progression of all-cause dementia

**DOI:** 10.1192/j.eurpsy.2022.458

**Published:** 2022-09-01

**Authors:** M. Bray, B. Bryant, A. Esagoff, L. Richey, C. Rodriguez, A. Krieg, C.M. Cullum, C. Lobue, Z. Ismail, M. Peters

**Affiliations:** 1 Johns Hopkins University School of Medicine, Department Of Psychiatry And Behavioral Sciences, Baltimore, United States of America; 2 University of Texas Southwestern, Department Of Psychiatry, Dallas, United States of America; 3 University of Calgary, Department Of Psychiatry, Calgary, Canada

**Keywords:** Dementia, neurodegeneration, traumatic brain injury, mild behavioral impairment

## Abstract

**Introduction:**

Traumatic brain injury (TBI) may alter dementia progression, although co-occurring neuropsychiatric symptoms (NPS) have received less attention. The mild behavioral impairment (MBI) construct relates NPS to underlying neural circuit disruptions, representing an important area of inquiry regarding TBI and dementia.

**Objectives:**

(1) to examine the influence of prior TBI history (preceding study enrollment) on MBI incidence in all-cause dementia (prior to dementia diagnosis, i.e. MBI’s original definition) and (2) to utilize MBI domains as a construct for examining the influence of TBI on related NPS across the course of dementia onset and progression.

**Methods:**

Using National Alzheimer’s Coordinating Center data, individuals progressing from normal cognition to all-cause dementia over 7.6±3.0 years were studied to estimate MBI incidence and symptom domains in 124 participants with prior TBI history compared to 822 without.

**Results:**

Moderate-severe TBI was associated with the social inappropriateness MBI domain (OR_adj._=4.034; *p*=0.024) prior to dementia onset, and the abnormal perception/thought content domain looking across dementia progression (HR_adj._=3.703, *p*=0.005). TBI (all severities) was associated with the decreased motivation domain looking throughout dementia progression (HR_adj._=1.546, *p*=0.014).

**Conclusions:**

TBI history is associated with particular MBI domains prior to onset and throughout progression of dementia. Understanding TBI’s impact on inter-related NPS may help elucidate underlying neuropathology.

**Disclosure:**

No significant relationships.

